# Total Knee Arthroplasty in Patients with Spinal Cord Injury: Impact on Medical Complications, Hospital Costs and Length of Stay

**DOI:** 10.5435/JAAOSGlobal-D-22-00145

**Published:** 2023-04-06

**Authors:** Senthil Sambandam, Naga Suresh Cheppalli, Anil Menedal, Tejas Senthil, Vishaal Sakthivelnathan, Varatharaj Mounasamy

**Affiliations:** From the University of Texas Southwestern, Dallas, TX (Dr. Sambandam); Dallas VAMC, Dallas, TX (Dr. Sambandam and Dr. Mounasamy); University of New Mexico School of Medicine, Albuquerque, NM (Dr. Cheppalli); VAMC, Albuquerque, NM (Dr. Cheppalli); Salem VA Medical Center, Salem, VA (Dr. Menedal); Carroll High School, Southlake, TX (Senthil); School of Medicine, University of Texas Medical Branch, Galveston, TX (Sakthivelnathan); and the Department of Orthopedics, University of Texas Southwestern, Dallas, TX (Dr. Mounasamy).

## Abstract

Patients with spinal cord injury (SCI) with degenerative joint disease of the knee may require total knee arthroplasty (TKA). This study examines the demographic and immediate postoperative outcomes of patients with SCI who undergo TKA.

**Methods:**

Admissions data for TKA and SCI were analyzed from the National Inpatient Sample database using *International Classification of Diseases, 10th Revision, Clinical Modification* diagnosis codes. An extensive array of preoperative and postoperative variables was compared among SCI TKA patients and non-SCI TKA patients. An unmatched and matched analysis using a 1:1 propensity match algorithm was conducted to compare the two groups.

**Results:**

Patients with SCI tend to be younger and have a 7.518 times greater risk of acute renal failure, 2.3 times greater risk of blood loss, and higher risk of local complications, including periprosthetic fracture and prosthetic infection. The average length of stay in the SCI cohort was 2.12 times greater, with a 1.58 times higher mean total incurred charge than the non-SCI group.

**Conclusion:**

SCI is associated with an increased risk of acute renal failure, blood loss anemia, periprosthetic fractures and infections, a longer length of stay, and greater incurred charges in TKA patients.

**Study Design:**

Retrospective study.

Spinal cord injury (SCI) is an uncommon but catastrophic condition with an estimated annual incidence of 50 per million population and an estimated prevalence of around 288,000 patients.^1,2,3,4,5^ Incomplete quadriplegia was noted to be the most common neurological lesion (40%); complete paraplegia was noted in nearly 20.4% of patients and complete quadriplegia in 11.5%. Patients with chronic SCI are also at risk of several secondary complications, including pressure ulcers, thrombotic events, urinary tract infections (UTIs), pyelonephritis, osteoporosis, and related fractures.^6,7,8,9,10,11,12,13,14^

Lower extremity joints, especially the hip, and knee problems have been reported in patients with SCI and are often related to osteoporosis, joint contracture, neuroarthropathy, and immobility-related cartilage degradation.^7,15,16,17,18,19,20,21,22,23^ Total knee arthroplasty (TKA) is a procedure indicated in SCI patients with end-stage joint destruction leading to pain and functional deterioration.

There is an absolute paucity of studies comparing the rates of complications in TKA patients with SCI with motor impairment compared with those without SCI.^24,25^ This study aims to assess the postoperative outcomes after TKA in patients with SCI using the National Inpatient Sample (NIS) database.

## Methods

### Database Description

This study was completed using the NIS database data from 2016 to 2019. The NIS database is a component of the Healthcare Cost and Utilization Project. It is the largest all-payer inpatient care database within the United States, with data from more than seven million hospital stays per year. The data encompass 20% of the hospitals in the United States and are verified using a quality assessment evaluation comparing data points with standardized normative values by an independent contractor.

Data was extracted from the NIS database. The database includes demographic data, length of stay, payment source, hospital charges, discharge status, comorbidities, etc. The 2016-2019 version uses the *International Classification of Diseases, 10th Revision, Clinical Modification/Procedure Coding System* (*ICD-10-CM/PCS*).

### Data Acquisition

This study was exempt from institutional review board approval because the data were deidentified and publicly available. Patients who underwent TKA were identified using the *ICD-10* procedural codes OSRC and OSRD. We identified patients with SCI using G82-related codes (Table 1).

**Table 1:** ICD-10 Codes Used

SCITKA	Obese Codes	Comorbidities Codes	Medical Complications Codes	Surgical Complications Codes
G82 G822 G8220 G8221 G8222 G8225 G82250 G82251 G82252 G82253 G82254	E660 E6601 E6609 E661 E662 E668 E669 Z6830 Z6831 Z6832 Z6833 Z6834 Z6835 Z6836 Z6837 Z6838 Z6839	Diabetes without complications E119 Diabetes with complications E1169 Tobacco-related disorder Z87891	Acute renal failure N170, N171, N172, N178, and N179 Myocardial infarction I2101, I2102, I2111, I2113, I12114, I12119, I2121, I12129, and I21A1 Blood loss anemia D62 Pneumonia J189, J159, and J22 Blood transfusion 30233N1 Pulmonary embolism I2602, I2609, I2692, and I2699 DVT I82401,I82402, I82403, I82409, I82411, I82412, I82413, I82419, I82421, I82422, I82423, I82429, I82431, I82432, I82433, I82439, I82441, I82442, I82443, I82449, I82491, I82492, I82493, I82499, I824Y1, I824Y2, I824Y3, I824Y9, I824Z1, I824Z2, I824Z3, and I824Z4	Periprosthetic fracture T84010A, T84011A, T84012A, T84013A, T84018A, T84019A, M9665, M96661, M96662, M96669, M96671, M96672, M96679, M9669, M9701XA, M9702XA, M9711XA, and M9712XA Periprosthetic dislocation T84020A, T84021A, T84022A, T84023A, T84028A, and T84029A Periprosthetic mechanical complications Periprosthetic fracture T84090A, T84091A, T84092A, T84093A, T84098A, and T84099A Periprosthetic infection T8450XA, T8451XA, T8452XA, T8453XA, T8454XA, and T8459XA Superficial SSI T8141XA Deep SSI T8142XA Wound dehiscence T8130XA, T8131XA, and T8132XA

DVT = deep vein thrombosis, ICD-10 = International Classification of Diseases, 10th Revision, SCITKA = spinal cord injury and underwent total knee arthroplasty, SSI = surgical site infection

Preoperative variables included (1) age, (2) sex, (3) elective versus nonelective admission, (4) diabetes without complications, (5) tobacco use disorder, and (6) obesity (Table 1). Postoperative medical and surgical outcomes aggregated from the NIS database include (1) length of stay, (2) total incurred charges, (3) mortality, (4) elective versus nonelective admission, (5) acute renal failure, (6) myocardial infarction, (7) blood loss anemia, (8) pneumonia, (9) pulmonary embolism, (10) deep vein thrombosis (DVT), (11) periprosthetic fracture, (12) periprosthetic dislocation, (13) periprosthetic mechanical complication, (14) periprosthetic infection, (15) superficial surgical site infection, (16) deep surgical site infection, (17) wound dehiscence, (18) cardiac arrest and ventricular fibrillation, and (19) blood transfusion (Table 1). These complications are representative of the most common complications of TKA available on the NIS database.

### Statistical Analysis

All statistical analyses were conducted using SPSS version 27.0 (IBM). Originally, descriptive statistics were used to aggregate patient demographic data. Unmatched and matched analyses were completed. A 1:1 propensity-matching algorithm using the preoperative variables was conducted. T-tests were used to analyze numerical variables. Chi square analyses were used when analyzing binomial variables. The Fisher exact test was used when the incidence values were less than 5. A *P* value of <0.05 was considered statistically significant for all tests. Odds ratios and their corresponding 95% confidence intervals for the surgical outcomes and complications were measured as a ratio of the incidence in the SCI and underwent TKA (SCITKA) group to the incidence in the SCITKA-negative control group.

## Results

A total of 558,371 patients who underwent TKA were identified using the NIS database; 558,311 patients had standard TKA (STKA) with no SCI, and 60 patients had SCITKA within that population.

### Demographic Data

On average, the patients in the SCITKA group, with a mean age of 62.18 years were younger than the patients in the control group, with a mean age of 66.72 years (*P* < 0.001). The SCITKA group tended to have fewer females, with the percentage of females being 53.33%, than the control group, with the percentage of females being 61.50% (*P* = 0.193). The incidence of tobacco-related disorders was less in the SCITKA group (5.00%) than in the control group (14.77%, *P* = 0.033). The other demographic variables were not significantly different among the two groups (Table 2).

**Table 2:** Patient Demographics of the Unmatched Cohort

Factor	SCITKA Group (n = 60)	Control Group (n = 558,311)	Odds Ratio (SCITKA Group/Control Group)	Odds Ratio 95% Confidence Interval	*P* Value
Mean age (SD) in years	62.18 (12.620)	66.72 (9.505)	—	—	<0.001
Sex (proportion of female)	32 (53.33%)	343,388 (61.50%)	0.715	0.431-1.187	0.193
Diabetes without complications (proportion of patients with diabetes)	5.00%	82,461 (14.77%)	0.304	0.095-0.970	0.033
Tobacco use disorder (proportion of users)	8.33%	88,364 (15.83%)	0.483	0.194-1.208	0.112
Obesity (proportion of obese)	17 (28.33%)	172,644 (30.92%)	0.883	0.504-1.549	0.664
Race White Black Hispanic Asian Native American	40 (66.6%) 11 (18.3%) 5% 6.6% 0	435,177 (77.9%) 44,828 (8.02%) 33,591 (6.01%) 8,237 (1.47%) 2,531 (0.45%)	—	—	<0.001

SCITKA = spinal cord injury and underwent total knee arthroplasty

### Unmatched Postoperative Outcomes Analysis

The mortality rate was greater in the SCITKA group (1.67%) than in the control group (0.03%, *P* < 0.001). The incidence of acute renal failure was greater in the SCITKA group (11.67%) than in thet control group (1.98%, *P* < 0.001). The incidence of blood loss anemia was greater in the SCITKA group (30.00%) than in the control group (15.32%, *P* = 0.002). The incidence of periprosthetic fracture was greater in the SCITKA group (3.33%) than in the control group (0.42%, *P* < 0.001). The incidence of periprosthetic infection was greater in the SCITKA group (6.67%) than in the control group (1.04%, *P* < 0.001). Other postoperative outcomes showed no significant differences among the two groups in the unmatched analysis (Table 3).

**Table 3:** Unmatched Analysis

Postoperative Variables	SCITKA Group (n = 60)	Control Group (n = 558,311)	Odds Ratio (SCITKA Group/Control Group)	Odds Ratio 95% Confidence Interval	*P* Value
Mortality	1.67%	197 (0.03%)	47.991	6.617-348.088	<0.001
Acute renal failure	11.67%	11,078 (1.98%)	6.524	2.966-14.353	<0.001
Myocardial infarction	0	109 (0.02%)	—	—	0.914
Blood loss anemia	18 (30.00%)	85,539 (15.32%)	2.369	1.364-4.115	0.002
Pneumonia	0	1,088 (0.19%)	—	—	0.732
Pulmonary embolism	0	1,238 (0.22%)	—	—	0.715
Deep vein thrombosis	0	1,262 (0.22%)	—	—	0.712
Periprosthetic fracture	3.33%	2,361 (0.42%)	8.120	1.982-33.264	<0.001
Periprosthetic dislocation	0	4,262 (0.76%)	—	—	0.497
Periprosthetic mechanical complication	1.67%	4,503 (0.81%)	2.085	0.289-15.048	0.456
Periprosthetic infection	6.67%	5,798 (1.04%)	6.807	2.467-18.777	<0.001
Superficial surgical site infection	0	26 (0.004%)	—	—	0.958
Deep surgical site infection	0	0.001%	—	—	0.977
Wound dehiscence	0	530 (0.09%)	—	—	0.811
Blood transfusion	3.33%	8,252 (14.78%)	2.299	0.561-9.412	0.234

SCITKA = spinal cord injury and underwent total knee arthroplasty

The average total incurred charges were higher for the SCITKA group, with a mean of $98,677.84 and a SD of $123,797.01, than in the control group, with a mean of $64,807.37 and a SD of $45,818.54 (*P* < 0.001) (Table 4). The average length of stay was higher in the SCITKA group, with a mean of 4.0 days and a SD of 4.3 days, than in the control group, with a mean of 2.3 days and a SD of 1.9 days (*P* < 0.001).

**Table 4:** Length of Stay and Cost of Care in the Unmatched and Matched Cohorts

Factor	Unmatched SCITKA	Unmatched Control	*P* Value	Matched SCITKA	Matched Control	*P* Value
Length of stay	4.00 (4.330)	2.35 (1.932)	<0.001	4.00 (4.330)	2.12 (1.061)	0.002
Hospital charges	98,677.84 (123,797.012)	64,807.37 (45,818.538)	<0.001	98,677.84 (123,797.012)	62,592.88 (36,983.588)	0.037

SCITKA = spinal cord injury and underwent total knee arthroplasty

### Matched Postoperative Outcomes Analysis

The 1:1 propensity match algorithm yielded 60 patients in the SCITKA group and 58 patients in the control group (Table 5). The incidence of acute renal failure was greater in the SCITKA group (11.67%) than in the control group (1.72%, *P* = 0.032). The incidence of periprosthetic dislocation was greater in the SCITKA group (0%) than in the control group (6.90%, *P* = 0.038). The other postoperative outcomes showed no significant differences among the two groups in the matched analysis (Table 6).

**Table 5:** Patient Demographics of the Matched Cohort

Factor	SCITKA Group (n = 60)	Control Group (n = 58)	Odds Ratio (SCITKA Group/Control Group)	Odds Ratio 95% Confidence Interval	*P* Value
Mean age (SD) in years	62.18 (12.620)	63.93 (9.464)	—	—	0.398
Sex (proportion of female)	32 (53.33%)	31 (53.44%)	0.995	0.483-2.052	0.990
Diabetes without complications (proportion of patients with diabetes)	5.00%	5.17%	0.965	0.187-4.987	0.966
Tobacco use disorder (proportion of users)	8.33%	6.90%	1.227	0.313-4.817	0.769
Obesity (proportion of obese)	17 (28.33%)	17 (29.31%)	0.907	0.430-2.115	0.344
Race White Black Hispanic Asian Native American	40 (66.6%) 11 (18.3%) 5% 6.6% 0	40 (68.9%) 11 (18.9%) 5.17% 6.89% 0	—	—	1.000
Age categorical <60 60-70 70-80 80-90	20 (33.3%) 24 (40%) 14 (23.3%) 3.33%	19 (32.7%) 23 39.6%) 14 (24.13%) 3.44%	—	—	1.000

SCITKA = spinal cord injury and underwent total knee arthroplasty

**Table 6:** Matched Analysis

Postoperative Variables	SCITKA Group (n = 60)	Control Group (n = 58)	Odds Ratio (SCITKA Group/Control Group)	Odds Ratio 95% Confidence Interval	*P* Value
Mortality	1.67%	1.72%	0.966	0.059-15.818	0.981
Acute renal failure	11.67%	1.72%	7.528	0.896-63.249	0.032
Myocardial infarction	0	0	^a^	^a^	^a^
Blood loss anemia	18 (30.00%)	12 (20.00%)	1.643	0.708-3.812	0.246
Pneumonia	0	0	^a^	^a^	^a^
Pulmonary embolism	0	0	^a^	^a^	^a^
Deep vein thrombosis	0	0	^a^	^a^	^a^
Periprosthetic fracture	3.33%	0	0.500	0.417-0.600	0.161
Periprosthetic dislocation	0	6.90%	0.474	0.390-0.575	0.038
Periprosthetic mechanical complication	1.67%	1.72%	0.966	0.059-15.818	0.981
Periprosthetic infection	6.67%	1.72%	4.071	0.441-37.567	0.183
Superficial surgical site infection	0	0	^a^	^a^	^a^
Deep surgical site infection	0	0	^a^	^a^	^a^
Wound dehiscence	0	0	^a^	^a^	^a^
Blood transfusion	3.33%	0	0.5	0.417-0.600	0.161

SCITKA = spinal cord injury and underwent total knee arthroplasty

a Because incidence was 0 in one or both groups, odds ratios and significance values could not be calculated.

The average total charges were higher in the SCITKA group (with a mean of $98,677.84 and a SD of $123,797.012) than in the control group (with a mean of $62,592.88 and a SD of $36,983.588) (*P* = 0.037). The average length of stay was higher in the SCITKA group, with a mean of 4.00 days and a SD of 4.330 days, than in the control group, with a mean of 2.12 days and a SD of 1.061 days (*P* = 0.002) (Table 4).

## Discussion

Our study showed that patients with paraplegia tend to be younger than those in the control group. Our study also revealed that after matching for patient demographics, patients with paraplegia are at almost 7 times higher risk of acute renal failure and the risk of blood loss anemia was 2.3 times higher; however, the risk of cardiac and pulmonary complications are not markedly different. Our study found that patients with paraplegia stayed twice as long and incurred approximately 50% higher costs than the control group. They were also at higher risk of local complications, including periprosthetic fracture and prosthetic infection.

Our study showed that the average age of the patients undergoing TKA was 62.18 years. There are only two case reports on patients with paraplegia undergoing TKA: the age of the patient in the study by Koubaa et al was 42 years and the age of the patient in the study by Zietek et al was 47 years.^24,25^ By contrast, the mean age of our patient population is older than the case reports and may be related to the large sample size.

Bozic et al^26^ analyzed the risk factors of revision after primary TKA in Medicare patients and noted that the prevalence of patients with paraplegia/hemiplegia in their study was 0.4%. The prevalence in our study was 0.0001%. The low prevalence noted in our study was probably related to the exclusion of patients with hemiplegia patients in our study because the patients with hemiplegia were older and associated with multiple comorbidities and represent different patient demographics compared with the patients with paraplegia.

Periprosthetic infection risk had been reported to be higher in the SCITKA patients because of the higher prevalence of UTI in these patients.^9,12,14,24,26^ Our study showed that the risk of periprosthetic joint infection was almost 4 times higher in the SCITKA patients, but it failed to show statistical significance. It is not uncommon for PP to have pressure ulcers that fail to heal after several years of treatment. In addition, the nutritional status of these patients in the setting of immobility and recurrent UTIs is not well-known. Inabathula et al^27^ reported extended antibiotic prophylaxis in high-risk patients. However, they failed to include SCITKA patients in their study. It may be reasonable to consider extended antibiotic prophylaxis in the PP high-risk patients undergoing TKA.

None of the patients with SCITKA had DVT or pulmonary embolism. However, the risk of DVT in chronic SCI is reported to be between 10 and 30%.^6,28,29^ Additional studies are needed to ascertain the risk of thrombotic complications after TKA in these high-risk patients and decide on the duration and strength of anticoagulation medications.

Several authors have reported the prevalence of osteoporosis in patients with paraplegia. The cumulative lifetime fracture risk in people with paraplegia is around 40%.^7,19^ The patient in the study by Zietek had a plate around the proximal tibia that required removal 3 months before the TKA.^25^ There was a 3.3% risk of periprosthetic fracture in the SCITKA group to 0% in the matched control group. Hence, surgeons conducting TKA in patients with PP should have fixation devices to deal with intraoperative fractures and consider cemented and stemmed implants to reduce stress in the metaphyseal region, thereby reducing the risk of periprosthetic fracture.

The average cost for SCITKA patients was approximately 50% higher. We do not have any other study to compare our findings. In the case reported by Zietek^25^ et al, totally stabilized implant with both a stemmed tibia and femur was utilized. Cost increases may be because of surgeons using more constrained implants for these patients. We also noted that SCITKA patients had higher medical and surgical complications and stayed longer, contributing to the increased cost.

Patients with spinal cord injuries can have varying levels of functionality based on their injury level and completeness of the injury. The American Spinal Injury Association has classified impairment into five classes based on sensory and motor status. Because there are only two cases reported in the literature reporting the outcome of TKA in patients with SCI, this database study can provide some information on immediate complications involved after TKA in these subset of patients. Because there is an increased rate of complications involved in this group of patients, strong reasons, such as pain and progressive loss of independence from knee osteoarthritis, should be established before surgeons recommend TKA. This database does not provide other details such as completeness, level of spinal injury, American Spinal Injury Association grading, duration from the spinal injury, and level of functional status before the surgery. The type and frequency of complication might change based on the level and completeness of spinal injury, and future studies are needed to determine the ideal patients for TKA in this subset of population.

Our study has limitations that are inherent to most database studies. Most databases retrospectively collect data and report it using Current Procedural Terminology or *ICD* codes. This process leads to the loss of granularity of the information and unavailability of important data such as laboratory values and functional scores. In addition, the accuracy of the data collected depends on the ability of the database to translate the clinical information correctly, subjecting the database to reporting errors and *ICD* coding errors. Another limitation was that only 20% of hospitals in the United States were represented in the NIS database. However, considering the large database size and extensive reliance on complex *ICD* codes, it is safe to assume that this database is a fair representation of the population undergoing TKA at the national level.

In conclusion, surgeons conducting TKA in paraplegic patients should be wary of increased costs and surgical and medical complications and be prepared for intraoperative challenges and the possible need for a postoperative multidisciplinary team approach.
